# Supplementary material for: Patterny: A Troupe of Decipherment Helpers for Intrinsic Disorder, Low Complexity and Compositional Bias in Proteins
Source: Biomolecules. 2025 Sep 18;15(9):1332. doi: 10.3390/biom15091332 (PMC12467476; doi:10.3390/biom15091332)
Supplement: Supplementary file 1 [file biomolecules-15-01332-s001.zip › Suppl-Tables-S1+S2.pdf]

Suppl. Table S1: Parameter sets used for definition of Compositional Modules

fLPS parameter set (m, M, t)	Target Length (len=)*	Estimated proteome coverage (cov=)*
7, 11, 5.2e-05	10	5%
12, 16, 5.4e-06	20	5%
10, 20, 1.8e-05	20	10%
9, 30, 6.9e-04	20	25%
21, 25, 6.2e-09	50	5%
23, 33, 4.1e-07	50	10%
20, 50, 4.7e-05	50	25%
32, 36, 7.9e-14	100	5%
38, 48, 7.3e-10	100	10%
34, 74, 5.2e-07	100	25%
68, 78, 4.1e-18	250	10%
78, 128, 2.1e-11	250	25%

Suppl. Table S2: Top ten domain folds in ASTRALSCOP40 with significant repetitiveness (low interval entropy, *IE*)

**Count of protein folds**	**SCOPe fold code**	**Description**
21	c.10	leucine-rich repeat, LRR (right-handed beta-alpha superhelix)
19	c.1	triose phosphate isomerase beta/alpha-barrel
18	d.58	ferredoxin-like
15	d.211	beta-hairpin-alpha-hairpin repeat
15	a.118	alpha-alpha superhelix
14	b.69	7-bladed beta-propeller
13	c.37	P-loop containing nucleoside triphosphate hydrolase
13	b.1	immunoglobulin-like beta-sandwich
11	b.68	6-bladed beta-propeller
10	g.37	beta-beta-alpha zinc finger
